# Childhood temporal lobe epilepsy: correlation between electroencephalography and magnetic resonance spectroscopy: a case–control study

**DOI:** 10.1186/s13052-015-0138-2

**Published:** 2015-04-18

**Authors:** Seham FA Azab, Laila M Sherief, Safaa H Saleh, Mona M Elshafeiy, Ahmed G Siam, Wafaa F Elsaeed, Mohamed A Arafa, Eman A Bendary, Hanan S Sherbiny, Rabab M Elbehedy, Khalid A Aziz

**Affiliations:** Faculty of Medicine, Zagazig University, 18 Omar Bin Elkhattab St, Al Qawmia, Zagazig City, Sharkia Governorate Egypt

**Keywords:** Temporal lobe epilepsy, Electroencephalography, Magnetic resonance spectroscopy, Children

## Abstract

**Background:**

The diagnosis of epilepsy should be made as early as possible to give a child the best chance for treatment success and also to decrease complications such as learning difficulties and social and behavioral problems. In this study, we aimed to assess the ability of magnetic resonance spectroscopy (MRS) in detecting the lateralization side in patients with Temporal lobe epilepsy (TLE) in correlation with EEG and MRI findings.

**Methods:**

This was a case–control study including 40 patients diagnosed (clinically and by EEG) as having temporal lobe epilepsy aged 8 to 14 years (mean, 10.4 years) and 20 healthy children with comparable age and gender as the control group. All patients were subjected to clinical examination, interictal electroencephalography and magnetic resonance imaging (MRI). Proton magnetic resonance spectroscopic examination (MRS) was performed to the patients and the controls.

**Results:**

According to the findings of electroencephalography, our patients were classified to three groups: Group 1 included 20 patients with unitemporal (lateralized) epileptic focus, group 2 included 12 patients with bitemporal (non-lateralized) epileptic focus and group 3 included 8 patients with normal electroencephalography. Magnetic resonance spectroscopy could lateralize the epileptic focus in 19 patients in group 1, nine patients in group2 and five patients in group 3 with overall lateralization of (82.5%), while electroencephalography was able to lateralize the focus in (50%) of patients and magnetic resonance imaging detected lateralization of mesial temporal sclerosis in (57.5%) of patients.

**Conclusion:**

Magnetic resonance spectroscopy is a promising tool in evaluating patients with epilepsy and offers increased sensitivity to detect temporal pathology that is not obvious on structural MRI imaging.

## Background

The overall incidence of new-onset epilepsy in children ranges from 33 to 82 per 100,000 children per year, and approximately half- to two-thirds of these children have focal-onset seizures [1]. However, the exact incidence of Temporal lobe epilepsy (TLE) is not known, as the specific lobe of onset is not specified in most incidence studies.

Symptoms of epilepsy may be difficult to recognize in children and adolescents, as not all seizures involve obvious convulsions. Sometimes symptoms are far more subtle, and these “hidden signs” may appear to fall within the range of normal childhood behavior, or in an adolescent, may be misinterpreted as psychological problems [2].

Epilepsy of temporal lobe origin is characterized by considerable heterogeneity in clinical history, ictal symptoms, electroencephalographic characteristics and neuroimaging finding [3].

Temporal lobe epilepsy (TLE) with seizure onset from the mesial temporal lobe structure is recognized as a syndrome of mesial temporal lobe epilepsy (MTLE) and it comprises the majority of cases of epilepsy refractory to pharmacotherapy [4].

In temporal lobe epilepsy the interictal scalp electroencephalography (EEG) may show the following: No abnormality, slight or marked asymmetry of the background activity, temporal spikes; sharp waves and/or slow waves, unilateral or bilateral, synchronous or asynchronous, these findings are not always confined to the temporal region [5].

On magnetic resonance images (MRI), mesial temporal sclerosis displays hippocampal atrophy, prolonged T2, and structural distortion. These changes may be assessed both qualitatively or by means of hippocampal volumetry and T2 relaxometry, which increase MR imaging sensitivity, even after quantitative studies, about 20% of patients with TLE have negative structural MR images [6]. Hydrogen spectroscopic imaging depicts the anatomic distribution of metabolite signals from N-acetylaspartate (NAA), creatine (Cr) and choline (Cho)-containing compounds, NAA is of major interest due to its predominantly neuronal distribution, reduction of NAA signal is usually interpreted as loss or dysfunction of neurons [7] Previous studies have shown that interictal NAA is reduced in the ipsilateral mesial temporal lobe, assisting in the lateralization of TLE even in cases with negative structural MR images [8]. Proton magnetic resonance spectroscopy (^1^HMRS) has been proposed as a method for the preoperative evaluation of patients with medically intractable temporal lobe epilepsy [9,10]. In this study, we aimed to assess the ability of magnetic resonance spectroscopy (MRS) in detecting the lateralization side in patients with TLE in correlation with EEG and MRI findings.

## Methods

This was a case control study performed in Zagazig University hospital from January 2011to December 2014. The study included 40 children and adolescents diagnosed (clinically and by EEG) as having temporal lobe epilepsy while attending Pediatric outpatient neurology clinic in Zagazig University Hospital. The age of the patients ranged from 8 to 14 years (mean, 10.4 years). Twenty healthy children of comparable age and gender served as a control group. The diagnosis of TLE was based on clinical history and seizures description according to the International League Against Epilepsy (ILAE) 1989 [5] as it defined TLE as a localization related epilepsy with typical clinical and EEG characteristic and it includes, simple partial, complex partial, secondary generalized or a combination of both.

Out of 40 patients 11 patients presented with simple partial seizures which was recognized by maintained conscious level and absence of automatism. Nine of them had simple motor seizures and the remaining two patients had simple sensory seizures (tingling and numbness). On the other hand 16 patients presented to us with features of complex partial seizures with automatic behavior and impaired consciousness. Video recordings of the parents have helped us to establish the diagnosis of these patients. The remaining 13 patients had partial seizures with secondary generalization (the onset of motor fits were mostly reported to start from the angle of the mouth or the upper limb).

Patients were excluded if the imaging study (by MRI) revealed any focal abnormalities other than mesial temporal sclerosis (MTS). So that patients included in the study are having temporal lobe epilepsy due to mesial temporal sclerosis or unknown cause.

Then we divided our TLE patients according to EEG finding into 3 groups;

- **Group 1** (n = 20): patients with EEG finding lateralized to one temporal lobe (unitemporal) and their mean age was 10.2 ± 2.6 years.
- **Group 2** (n = 12): patients without lateralization of the focus by EEG (bitemporal) with a mean age of 9.8 ± 1.7 years.
- **Group 3** (n = 8): patients clinically diagnosed as TLE with normal EEG and their mean age was 11 ± 2.9 years.

All patients and controls included were subjected to proper history taking, thorough clinical neurological examination and MRS examination. Video scalp EEG and MRI brain were performed to the patients.

Electroencephalography (EEG) was done to all patients using the Stellate Harmonie system. This system has a standard number of 28 channels. During this procedure, the EEG is recorded for a prolonged period, accompanied by continuous closed-circuit video observation. The digitalized EEG and recorded behavior are displayed simultaneously, allowing point-to-point correlations of recorded events and any accompanying electrographic changes.

During video-EEG monitoring, the patient wears an EEG transmitter connected to coaxial cable. Wall-mounted video cameras provide continuous behavioral observation. Both EEG and video signals are transmitted to the EEG recording device, The EEG signal and video are displayed simultaneously for on-line observation, and both are recorded on hard drive or DVDs.

The entire duration of the EEG evaluation was analyzed, a sharp wave was considered epileptiform when it had a sharp morphology, duration of < 200 ms, and was clearly distinct from EEG background. Unitemporal interictal discharges (IEDs) required a minimum of 80% of lateralization. In patients with <80% of interictal EEG onset lateralized to one side were considered bitemporal IEDs [7].

All patients underwent conventional MRI study at 1.5-Tesla MR units (Achiva; Philips Medical Systems, Cleveland, Ohio). Conventional axial and sagittal T1-weighted images (TR 500 ms/TE 15 ms/slice thickness 5 mm/slice gap 1.5 nun), axial FLAIR (TR 6000 ms/TE 120 ms/TI 2000 ms thickness 5 mm/slice gab 1 mm). Thin coronal sections were obtained for temporal lobes, included T2WI (TR 3000 ms/TE 80 ms) and FLAIR images (TR 6000 ms/TE120ms with TI 2000).

Single-voxel proton MR spectroscopy was carried out immediately after MR imaging with an 8 cm^3^ voxel (2X2X2 cm) positioned over the mesial temporal lobe at thin coronal T2 WI or FLAIR, including a part of the hippocampus. A point-resolved spectroscopy (PRESS) with chemical-shift selective (CHESS) water suppressed spectroscopic images were acquired. The acquisition parameters used were: TR of 1000, TE of 35 ms, a field of view of 240 × 240 mm, and 32 × 32 phase encodes with a slice thickness of 1 cm. An automatic and operator-nondependent processing scheme was achieved to analyze the spectra recorded for our study. The signal intensity of various metabolite peaks was evaluated in every voxel, using integrals of each peak as a measure of its intensity. Three resonances of the important metabolites were identified: NAA at 2.02 ppm, Cr at 3.02 ppm, and Cho at 3.22 ppm. The relative ratios of NAA to Cr were estimated for both temporal lobes of each patient and control group.

### Ethics

Informed parental consent was obtained to be eligible for enrollment into the study. The study was done according to the rules of the Local Ethics Committee of Faculty of Medicine, Zagazig University, Egypt.

### Statistical methods

SPSS version 19 was used for data analysis. The data are expressed as the mean ± SD or median (min-max) where appropriate. Test selection was based on evaluating the variables for normal distribution using the *Shapiro-Wilk* test. If the variables had a normal distribution, *Student’s t-test* was used. If the variable did not have a normal distribution, the analysis was done using the *Mann–Whitney U* test. Categorical data were evaluated by *Pearson’s chi-square* test. The correlations between variables were performed by *Pearson’s Correlation* test. *P* < 0.05 was considered significant.

## Results

The demographic characteristic of the 3 groups of patients were illustrated in (Table 1). Group 1 included 20 patients (11 males and 9 females) with EEG finding lateralized to one temporal lobe (unitemporal) and their mean age was 10.2 ± 2.6 years. Group 2 included 12 patients (6 males and 6 females) without lateralization of the focus by EEG (bitemporal) with a mean age of 9.8 ± 1.7 years. Group 3 included 8 patients (5 males and 3 females) clinically diagnosed as TLE with normal EEG and their mean age was 11 ± 2.9 years. There was no significant difference between the 3 groups as regards age, gender, duration of epilepsy or previous insult (*P* > 0.05), respectively.

**Table 1:** **Demographic data of the patients**

	**Group 1**	**Group 2**	**Group 3**	**P**
	**n = 20**	**n = 12**	**n = 8**	
**Age (years)**	10.2 ± 2.6	9.8 ± 1.7	11 ± 2.9	0.88
**Gender (M/F)**	11/9	6/6	5/3	0.84
**Duration of epilepsy (years)**	2.3 ± 0.8	1.9 ± 0.6	2.1 ± 1	0.8
***Previous insult:***				
***Febrile convulsions***	4	2	1	0.77
***CNS infection***	2	1	0	0.56

*P* value < 0.05 indicates a significant difference.

When we compared the metabolic ratio (NAA/Cr ratio) between the 3 groups of patients and the control group we found that there was a significant difference between the patients and the control groups with a decreased level in the patients groups (Table 2).

**Table 2:** **Metabolic ratio by MRS in the 3 groups of patients and control group**

	**Group 1**	**Group 2**	**Group 3**	**Control**	**P-value**
	**n = 20**	**n = 12**	**n = 8**	**n = 20**	
**NAA/Cr (Lt)**	1.68 ± 0.4	1.64 ± 0.6	1.63 ± 0.5	1.90 ± 0.26	0.04
***NAA/Cr (Rt)***	1.58 ± 0.4	1.59 ± 0.5	1.57 ± 0.48	1.99 ± 0.31	0.02

NAA/Cr: N-acetyl aspartate/creatin ratio, Lt: Left, Rt: Right. *P* value < 0.05 indicates a significant difference.

When we study the ability of ^1^HMRS and MRI to localize the side of epileptic focus in comparison with EEG, we found that, in the first group with unitemporal localized IEDs in EEG (20 patients) the epileptic focus was unilateral in 15 patients and bilateral with left or right predominance in 5 patients, in this group ^1^HMRS abnormalities (decreased NAA/Cr ratio) were lateralized in 19 out of 20 patients (95%) as it was unilateral in 16 patients and bilateral with left or right predominance in 3 patients and MRS was normal in 1 patient, while in this group MRI detected abnormalities with mesial temporal sclerosis (MTS) lateralized to one side in 11 (55%) patients out of 20 patients and the remaining 9 patients; 4 patients had nonlateralized lesions and 5 patients had normal MRI.

MRI abnormalities are solely related to mesial temporal sclerosis as other focal MRI abnormalities were excluded from the study. These MRI findings included reduced hipoocampal volume, increased T2 signal intensity with or without volume change and or loss of interdigitations of hippocampus.

The second group included 12 patients with bitemporal nonlateralized IEDs on EEG, we found that ^1^HMRS showed lateralization in 9 patients (75%) as it was unilateral in 6 patients and bilateral with one side predominance in 3 patients and the other 3 patients (25%) showed bitemporal metabolic abnormalities with no lateralization, meanwhile MRI showed abnormalities in 9 patients (75%) and it was normal in 3 patients (25%).

The third group of patients with TLE included 8 patients with normal EEG, we found that ^1^HMRS detected metabolic abnormalities in 7 patients (87.5%); 5 patients (62.5%) with unitemporal lateralized lesions and 2 patients (25%) with bitemporal metabolic abnormalities without lateralization, and one patient (12.5%) had normal MRS meanwhile MRI showed abnormalities lateralized to one side in 3 patients in this group (37.5%) and 2 patient (25%) had nonlateralized lesion and 3 patient (37.5%) had normal MRI (Table 3).

**Table 3:** **Comparison between EEG,** ^**1**^ **HMRS and MRI for lateralization of the side of the lesions**

		**Group 1**	**Group 2**	**Group 3**
		**(n = 20)**	**(n = 12)**	**(n = 8)**
EEG lateralization *N* (%)	**Lateralized**	20(50%)	-	-
	*Strictly lateralized*	15		
	*Lateralized with side Predominance*	5		
	**Non lateralized**	-	12(30%)	-
	Normal EEG	-	-	8(20%)
^1^HMRS lateralization *N* (%)	**Lateralized**	19(95%)	9(75%)	5(62.5%)
	*Strictly lateralized*	16		
	*Lateralized with side Predominance*	3		
	Non lateralized	-	3(25%)	2(25%)
	Normal MRS	1(5%)	-	1(12.5%)
MRI lateralization *N* (%)	Lateralized	11(55%)	9(75%)	3(37.5%)
	Non lateralized	4(20%)	-	2(25%)
	Normal MRI	5(25%)	3(25%)	3(37.5%)

^1^HMRS: Magnetic resonance spectroscopy, MRI: Magnetic resonance imaging, n: Number.

Out of 40 patients with TLE; EEG detected lateralization of IEDs in 20 patients (50%) while ^1^HMRS detected metabolic abnormalities lateralized to one side in 33 patients (82.5%) and MRI detected lateralized abnormalities in 23 patients (57.5%) (Table 3).

**Figure 1:**
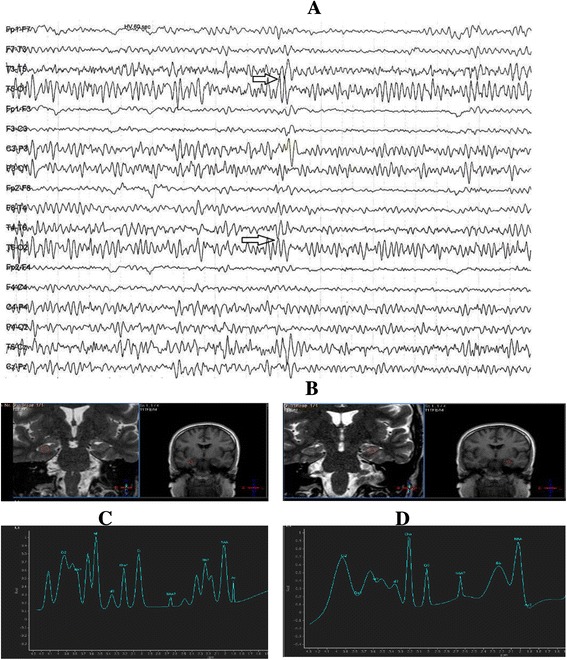
A patient with temporal lobe epilepsy; EEG showed bitemporal interictal discharges without lateralization **(A)**, both mesial temporal lobes showed abnormal high signal with atrophic changes at MRI **(B)**, while at MRS showed abnormal decrease of NAA/Cr ratio at both temporal lobes more evident at right side **(C)**, denoted right side lateralization, Left side NAA/Cr = 1.5 **(D)**. Right side NAA/Cr ratio = 1.1.

**Figure 2:**
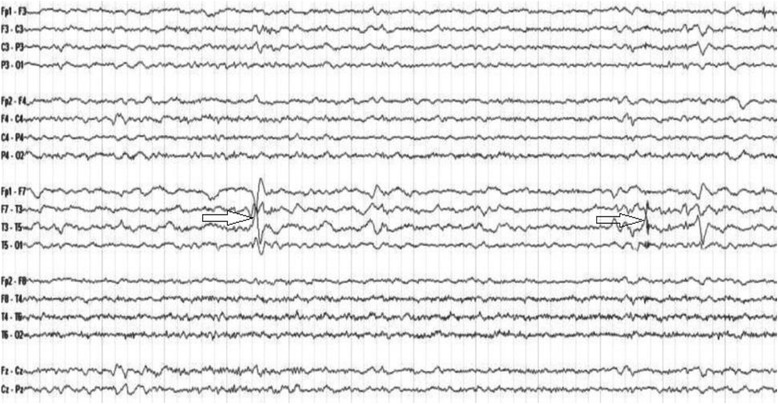
A patient with temporal lobe epilepsy: EEG showed left temporal spikes and sharp waves with phase reversal.

When we study the correlation between IEDs on EEG and metabolic changes by ^1^HMRS we found that there was a significant negative correlation between IEDs and NAA/Cr ratio (Table 4). IEDs was related to the metabolic changes but without strict related lateralization (Figures 1 and 2).

**Table 4:** **Correlation between** ^**1**^ **HMRS metabolites and interictal epileptiform discharges**

	**r**	**P**
**Lt IEDs vs Lt NAA/Cr**	−0.238	>0.05
**Rt IEDs vs Rt NAA/Cr**	−0.355	>0.05
***All IEDs vs all NAA*** **’** ***Cr***	−0.599	<0.05

Lt IEDs: Left interictal discharges; Rt IEDs: Right interictal discharges; Lt NAA/Cr: Left N-acetyl-aspartate/creatine, Rt NAA/Cr: Right N-acetyl-aspartate/creatine. *P* value < 0.05 indicates a significant difference.

## Discussion

In adults, TLE is characterized by a well described semiology that includes gastric auras, arrest of activity, automatisms, and altered consciousness, but in contrast, seizures in children have a much wider clinical spectrum depending on the age of the child [11]. In the management of temporal lobe epilepsy, exact lateralization of seizure focus with a noninvasive study is crucial, because surgical resection of the epileptogenic lesion results in good outcome and a failure to lateralize the focus with noninvasive examination may lead to an invasive study or to additional surgery for placement of intracranial electrodes, which may have potential risks [12].

However, many sources of temporal lobe epilepsy, including hippocampal sclerosis and developmental anomalies, may not be recognized, even by a variety of MR imaging techniques, including MR volumetry and measurement of T2 relaxation time [3]. Preliminary reports on proton MRS have noted that it is a promising neuroimaging modality and most MRS studies in patients with temporal lobe epilepsy have shown a decrease in NAA, and NAA/Cr ratio [13] and this result was in agreement with our results as we found that NAA/Cr ratio decreased significantly in patients with TLE than in controls.

Hammen and colleagues [14] reported that the role of NAA is not established in detail. First reports attributed NAA reduction to irreversible neuronal tissue degeneration and cell loss, but in later studies, alteration of NAA were also associated with neuronal dysfunction reflecting a reversible, dynamic state not principally characterized by tissue damage. Later studies focusing on neuronal dysfunction rather than cell death showed that NAA reduction is mainly based on mitochondrial pathway.

The aim of our study was to investigate whether proton MRS in patients with TLE can detects the side of epileptic focus (side of lateralization) in relation to EEG. We found that ^1^HMRS detected side of lateralization in (82.5%) of patients while MRI detected abnormalities in (57.5%) and EEG detected lateralization in (50%) of patients.

EEG detected lateralization in 20 patients (15 of them were strictly lateralized and 5 patients showed left or right predominance). MRI detected lateralization in 23 patients. HMRS detected lateralization in 33 patients (27 of them strictly lateralized) and so it has detected lateralization in 13 patients more than that detected by EEG study and in 10 patients over than those detected by MRI. This finding clarify that HMRS was more sensitive in detection of lateralization. We could explain that in these cases the MRS certainly adds something to the diagnosis, but not enough to induce a choice of surgical strategy.

The results of the EEG (its ability in detecting the epileptic focus) was in agreement with Ebnor and Hopp [15] as they reported that bitemporal spikes or sharp waves, maximal on the side of seizure origin, occur in 25% to 50% of patients with TLE. But in Foldvary and colleagues [16] study, they found that epileptic discharges are absent on serial recordings in 10% of patients with TLE. On the other hand, Blume [17] suggested that the lack of temporal lobe epileptic form activity of TLE in about three routine EEGs needs reassessment of the diagnosis.

As regarding the relation between ^1^HMRS and EEG, we found that there was a significant negative correlation between IEDs and NAA/Cr ratio and these results were concordant with most of the previous studies as; Garcia and colleagues [18] studied 16 patients with temporal lobe epilepsy by EEG and ^1^HMRS and they found that decreasing NAA correlated with increased spike frequency, and Sarles and colleagues [19] studied 14 patients with frontal and temporal lobe epilepsy and they found that there was an overall trend in which spike frequency and NAA/Cr ratio were correlated inversely, although the results did not reach statistical significance, the trend was obvious.

Also, Maton and colleagues [20] in their study on 31 patients with TLE found that lateralized ^1^HMRS based hipocompal abnormalities were recorded in 95% of the patients with unilateral interictal epileptiform abnormalities and they found that bilateral metabolic ^1^H MRS-based abnormalities were recorded 3 times more often than was bitemporal EEG spiking, and Hammen and colleagues [14] studied 14 patients with TLE and they found a significant negative correlation between NAA values and degree of IEDs in intensive video EEG recording and also there were a significant negative correlation between duration of seizures and NAA/Cr ratio.

While Park and colleagues [21] in their study of 34 patient with mesial TLE found that reduced NAA/Cr ratio were correlated to IEDs contralateral but not ipsilateral to the EEG focus. They interpreted these results as a triggering pathomechanism located contralateral to the underlying EEG focus propably generating independent contralateral IEDs.

In our study MRI detected changes in 23 (57.5%) out of 40 patients and 11 patients had normal MRI. Out of these 11 patients, MRS showed changes in 9 patients. This results were in agreement with Connelley and colleagues [22] in their study of 25 patients as they found that 19 patients (76%) were believed to have hipocampal sclerosis. Also Connelly and colleagues [23] in a different study found that MRS could detect metabolic abnormalities in patients with normal MRI and they concluded that MRS clearly provides an added value to MRI and enhance the sensitivity of global MR examination.

## Conclusion

MRS is a noninvasive neuroradiology technique. It is a promising tool in evaluating patients with epilepsy and offers increased sensitivity to detect mesial temporal pathology that is not obvious on structural MRI imaging. So, EEG and clinical data, in conjunction with magnetic resonance imaging (MRI) and magnetic resonance spectroscopy (MRS) results, help the localization of the epileptogenic zone in patients with TLE.

## Abbreviations

TLE: Temporal lobe epilepsy
MTLE: Mesial temporal lobe epilepsy EEG Electroencephalography
MRI: Magnetic resonance imaging
MRS: Magnetic resonance spectroscopy MTS mesial temporal sclerosis PRESS point-resolved spectroscopy
CHESS: Chemical-shift selective
^1^HMRS: Proton magnetic resonance spectroscopy IEDs inter ictal discharges
